# Direct uptake of sphingosine-1-phosphate independent of phospholipid phosphatases

**DOI:** 10.1016/j.jbc.2021.100605

**Published:** 2021-03-27

**Authors:** Hirotaka Goto, Masatoshi Miyamoto, Akio Kihara

**Affiliations:** Faculty of Pharmaceutical Sciences, Hokkaido University, Sapporo, Japan

**Keywords:** ceramide, channel, endothelial cell, erythrocyte, lipid, phosphatidylethanolamine, phospholipid phosphatase, sphingolipid, sphingosine-1-phosphate, transporter, BSA, bovine serum albumin, CERS, ceramide synthase, FBS, fetal bovine serum, HA, hemagglutinin, HDL, high-density lipoprotein, HUVEC, human umbilical vein endothelial cell, LPP, lipid phosphate phosphatase, MEDEP, mouse ES cell-derived erythroid progenitor line, MFSD2B, major facilitator superfamily domain containing 2B, PA, phosphatidic acid, PAP, phosphatidic acid phosphatase, PC, phosphatidylcholine, PE, phosphatidylethanolamine, Pi, orthophosphoric acid, PI, phosphatidylinositol, PLPP, phospholipid phosphatase, PS, phosphatidylserine, S1P, sphingosine-1-phosphate, Sph, sphingosine, S1PR, sphingosine-1-phosphate receptor, SPNS2, sphingolipid transporter 2, TKO, triple KO, UPLC, ultra–high performance liquid chromatography

## Abstract

Sphingosine-1-phosphate (S1P) is a lipid mediator that is relatively abundant in plasma and plays an important role in the vascular and immune systems. To date, the only known mechanism for removing S1P from plasma has been dephosphorylation by phospholipid phosphatases (PLPPs) on the surface of cells in contact with the plasma. However, there remains a possibility that PLPP-independent dephosphorylation or direct S1P uptake into cells could occur. To examine these possibilities, here we generated triple KO (TKO) HAP1 cells that lacked all PLPPs (*PLPP1–3*) present in mammals. In the TKO cells, the intracellular metabolism of externally added deuterium-labeled S1P to ceramide was reduced to 17% compared with the WT cells, indicating that most extracellular S1P is dephosphorylated by PLPPs and then taken up into cells. However, this result also reveals the existence of a PLPP-independent S1P uptake pathway. Tracer experiments using [^32^P]S1P showed the existence of a direct S1P uptake pathway that functions without prior dephosphorylation. Overexpression of sphingolipid transporter 2 (SPNS2) or of major facilitator superfamily domain containing 2B (MFSD2B), both known S1P efflux transporters, in TKO cells increased the direct uptake of S1P, whereas KO of *MFSD2B* in TKO cells reduced this uptake. These results suggest that these are channel-type transporters and capable of not only exporting but also importing S1P. Furthermore, we observed that erythroid cells expressing MFSD2B, exhibited high S1P uptake activity. Our findings describing direct S1P uptake may contribute to the elucidation of the molecular mechanisms that regulate plasma S1P concentration.

The lipid mediator sphingosine-1-phosphate (S1P) induces a variety of cellular responses, such as cell proliferation, upregulation or downregulation of cell motility, actin cytoskeleton rearrangement, and adherens junction assembly, through binding to the cell surface receptors (sphingosine-1-phosphate receptors 1 to 5 [S1PR1 to S1PR5]) (1, 2, 3). These actions of S1P are particularly important for embryonic vascular formation and the immune system. In sphingosine-1-phosphate receptorS1PR1 KO mice, vascular stabilization is impaired, resulting in embryonic lethality (4). In the immune system, S1P plays a pivotal role in the egress of T cells from the thymus and secondary lymphoid tissues (1, 2, 3). This function has been applied to the development of fingolimod, the therapeutic agent of multiple sclerosis (5, 6).

Sphingolipids are one of the major lipids that constitute eukaryotic cell membranes, together with glycerophospholipids and sterols. Sphingolipids are composed of a hydrophobic ceramide backbone, in which a long-chain base is amide bonded with a fatty acid, and a polar group (7). The major long-chain base in mammals is sphingosine (Sph), and S1P is produced intracellularly *via* phosphorylation of Sph by Sph kinases (1, 7, 8, 9). In degradation pathways, S1P is either dephosphorylated back to Sph by S1P phosphatases or cleaved by S1P lyase (1, 10, 11, 12, 13) (Fig. 1). In the former pathway, the generated Sph is reused for sphingolipid synthesis. In the latter pathway, the cleavage of S1P produces the fatty aldehyde *trans*-2-hexadecenal and phosphoethanolamine (10, 13). *Trans*-2-hexadecenal is converted to palmitoyl-CoA through oxidation, CoA addition, and saturation, followed by incorporation into lipids (mainly glycerolipids) or β-oxidation (7, 13, 14, 15, 16) (Fig. 1). Phosphoethanolamine is used for the synthesis of phosphatidylethanolamine (PE), one of the glycerophospholipids, after being converted to CDP-ethanolamine (13, 17, 18).

**Figure 1:**
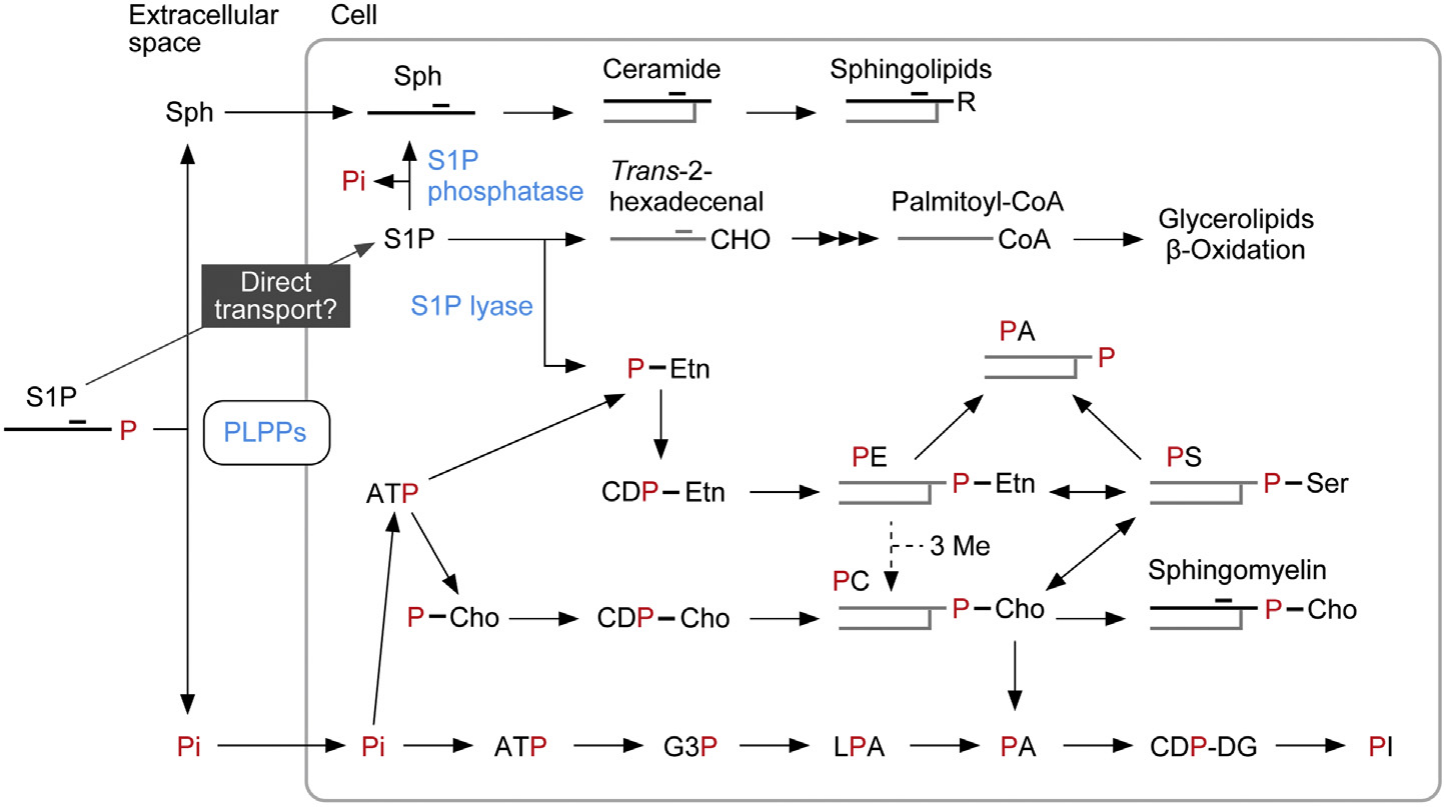
**S1P metabolic pathway.** The phosphate (P) group of S1P is illustrated in *red*, and lipids containing *red* P can be radiolabeled by [^32^P]S1P. S1P transported directly into cells is dephosphorylated by S1P phosphatases or cleaved by S1P lyase. The former reaction produces Sph, and the latter reaction produces *trans*-2-hexadecenal and phosphoethanolamine. Sph is metabolized to complex sphingolipids *via* ceramide. *Trans*-2-hexadecenal is metabolized to palmitoyl-CoA *via trans*-2-hexadecenoic acid and *trans*-2-hexadecenoyl-CoA and then subjected to lipid synthesis (mainly glycerolipids) or β-oxidation. Phosphoethanolamine is metabolized to PE *via* CDP-ethanolamine. PE can be converted to PS through a base-exchange reaction, and a fraction of the PS generated is further converted to PC through another base-exchange reaction. Alternatively, PE can be metabolized to PC by receiving three methyl groups, a reaction that occurs in the liver (18). A fraction of PC is used in a sphingomyelin-producing reaction, where phosphocholine is transferred to ceramide (52). Some of the PE and PC are degraded by phospholipase D to PA. Most extracellular S1P is dephosphorylated by PLPPs, generating Pi and Sph. Sph is rapidly imported into cells and metabolized to ceramides and then further to complex sphingolipids. After being transported into cells, Pi is converted to ATP, and then to phosphoethanolamine, phosphocholine, or glycerol 3-phosphate, followed by metabolism to glycerophospholipids. Cho, choline; CHO, aldehyde group; DG, diacylglycerol; Etn, ethanolamine; G3P, glycerol 3-phosphate; LPA, lysophosphatidic acid; Me, methyl group; PA, phosphatidic acid; PC, phosphatidylcholine; P-Cho, phosphocholine; PE, phosphatidylethanolamine; P-Etn, phosphoethanolamine; Pi, orthophosphoric acid; PI, phosphatidylinositol; PLPP, phospholipid phosphatase; PS, phosphatidylserine; R, polar group; S1P, sphingosine-1-phosphate; Sph, sphingosine.

Since S1P is an intermediate of the sole degradation pathway of sphingolipids, it is generated in all mammalian cells and tissues (1, 19). However, the intracellular S1P concentration is low because of the high S1P degradation activity in most cells (20, 21). On the other hand, the intracellular S1P concentration in platelets is high, since platelets have high Sph kinase activity and do not have S1P lyase (22, 23, 24, 25). In addition, erythrocytes store relatively high amounts of S1P (22, 25). Although erythrocytes have low Sph kinase activity, they have neither S1P lyase nor S1P phosphatases (25).

To date, two S1P efflux transporters, sphingolipid transporter 2 (SPNS2) and major facilitator superfamily domain containing 2B (MFSD2B), have been identified (26, 27, 28). MFSD2B is expressed in erythrocytes and platelets (27, 28), whereas SPNS2 is present in endothelial cells (29, 30). Transporters are classified into two types: pumps for active transport and channels for passive transport. Pump-type transporters move substances using energy, such as ATP hydrolysis or an ion concentration gradient. In contrast, channel-type transporters allow the movement of substances depending on the concentration gradient of the substances themselves. It currently remains unclear whether the S1P transporters SPNS2 and MFSD2B are pumps or channels.

S1P exists in plasma at relatively high concentration (hundreds of nanomolar to micromolar) bound to albumin or high-density lipoprotein (HDL) (1, 22, 27, 31, 32, 33). The major source of plasma S1P is erythrocytes (25, 27, 34, 35), although endothelial cells also contribute (29, 30). S1P release from platelets is dependent on platelet activation (36), and platelet contribution to plasma S1P is low (35). However, platelets do contribute to serum S1P, which has a higher concentration than plasma S1P (22, 31, 32). S1P stored in platelets is thought to play a role in the repair of endothelial vessels after injury (37).

S1P lyase and S1P phosphatases are localized in the endoplasmic reticulum and involved in the degradation of intracellular S1P (12, 24). On the other hand, the degradation of extracellular S1P, such as the S1P in plasma, is catalyzed by the phospholipid phosphatase (PLPP)/lipid phosphate phosphatase (LPP) family (38, 39). Mammals have three PLPP members, PLPP1 (also known as LPP1 or phosphatidic acid phosphatase [PAP]-2a), PLPP2 (LPP2/PAP-2c), and PLPP3 (LPP3/PAP-2b). These dephosphorylate not only S1P but also other phosphorylated lipids, such as lysophosphatidic acid, phosphatidic acid (PA), and ceramide 1-phosphate (38, 39). The efficiency of extracellular S1P uptake into cells is low, whereas that of Sph is high (40). Therefore, extracellular S1P is considered to be taken into cells after being dephosphorylated to Sph by the PLPP family; PLPP1 was shown to be involved in this process in lung endothelial cells (41). However, it is unclear what proportion of extracellular S1P is taken up by cells after PLPP-catalyzed dephosphorylation. It also remains possible that some S1P is transported into cells directly (without dephosphorylation) by unknown transporter(s).

In the present study, to clarify the exact contribution of PLPPs to the overall uptake of extracellular S1P into cells, we created *PLPP1–3* triple KO (TKO) cells. Although the contribution of the PLPP-dependent pathway was high, a PLPP-independent transport pathway directly importing S1P without dephosphorylation did exist. The direct S1P uptake activity was enhanced by overexpressing SPNS2 or MFSD2B and decreased by KO of *MFSD2B* in TKO cells. Furthermore, S1P uptake activity was also observed in erythroid cells, where MFSD2B is highly expressed. From these results, we speculate that plasma S1P concentration is regulated not only by S1P release from erythrocytes/endothelial cells and dephosphorylation by PLPPs but also by the direct uptake of S1P into cells in contact with plasma (especially erythrocytes). Furthermore, our results suggest that SPNS2 and MFSD2B are channel-type transporters that transport S1P both inward and outward, depending on the intracellular and extracellular S1P concentrations.

## Results

### A PLPP-independent S1P uptake pathway exists

**Figure 2:**
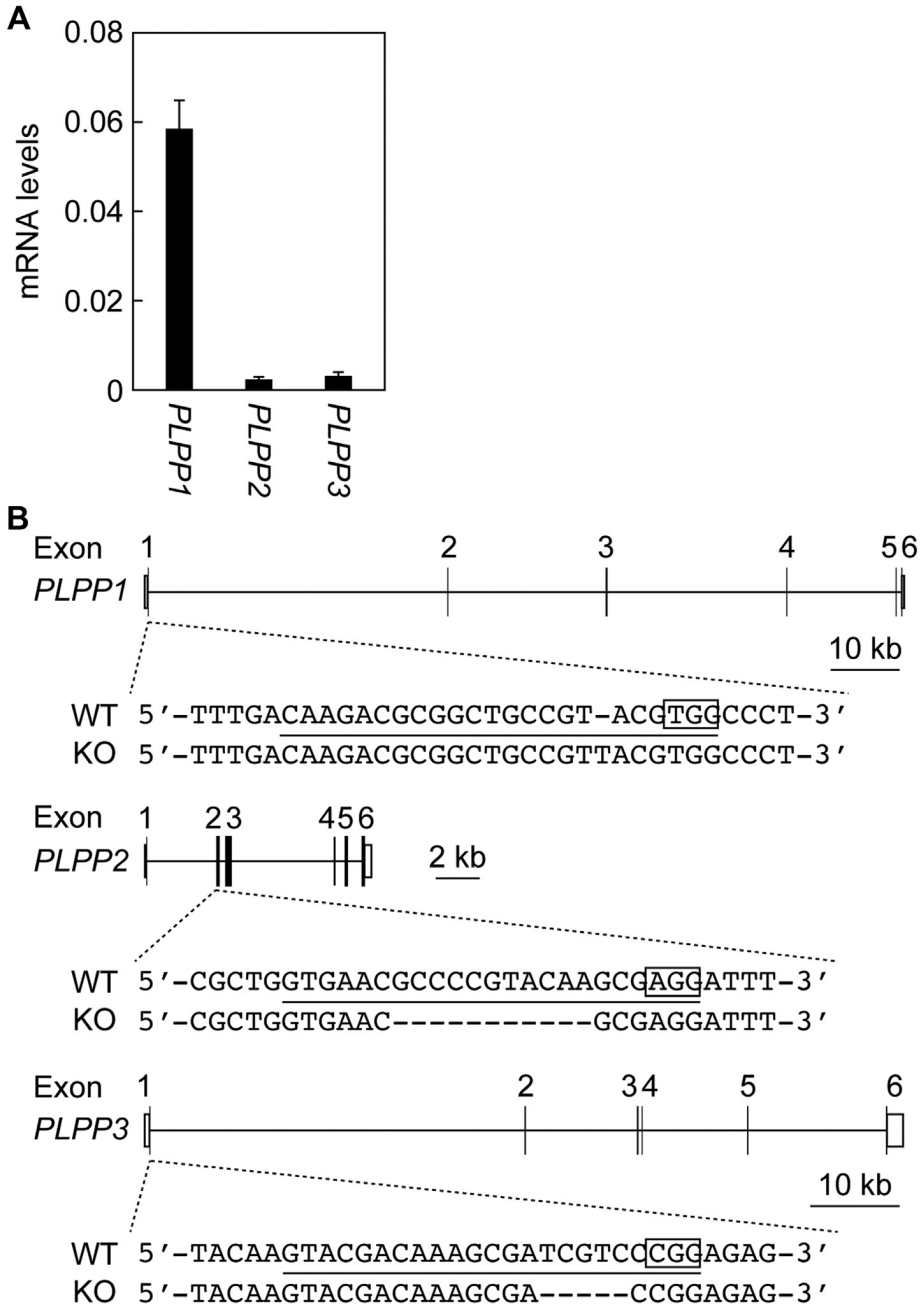
**Construction of *PLPP1–3* triple KO cells.***A*, total RNA prepared from HAP1 cells was subjected to quantitative real-time RT-PCR using specific primers for *PLPP1*, *PLPP2*, *PLPP3*, and *GAPDH*. Values represent the means ± SD and indicate the quantities relative to that of *GAPDH* from three independent reactions. *B*, *PLPP1–3* triple KO cells were constructed from HAP1 cells using the CRISPR/Cas9 system. The genome structures (*coding sequences*, *black boxes*; untranslated regions, *white boxes*), the location and types of mutations, protospacer adjacent motifs (*enclosed*), and the guide RNA sequences (*underlined*) for *PLPP1–3* are shown. PLPP, phospholipid phosphatase.

To investigate the contribution of the PLPP-dependent dephosphorylation pathway to S1P uptake, *PLPP1–3* TKO cells were generated using the CRISPR/Cas9 system from the near-haploid human cells, HAP1. A quantitative real-time RT-PCR revealed that the expression levels of *PLPP1* were much higher than those of *PLPP2* and *PLPP3* in HAP1 WT cells (Fig. 2*A*). The generated *PLPP1–3* TKO cells contained a 1-bp insertion in *PLPP1*, an 11-bp deletion in *PLPP2*, and a 5-bp deletion in *PLPP3* (Fig. 2*B*).

**Figure 3:**
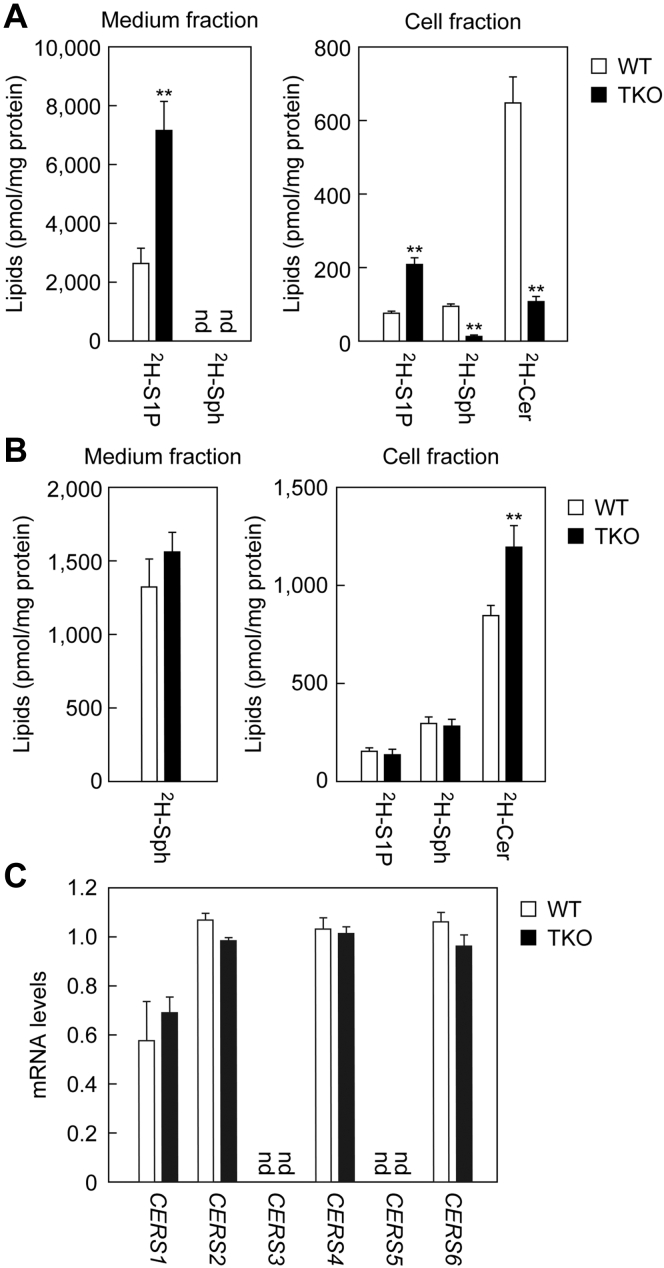
**Existence of an S1P uptake mechanism independent of PLPPs.***A* and *B*, WT and *PLPP1–3* TKO HAP1 cells were incubated with 5 μM ^2^H_7_-S1P (*A*) or ^2^H_7_-Sph (*B*) for 2 h. After separation into medium and cell fractions by centrifugation, lipids were extracted from each, and ^2^H_7_-S1P, ^2^H_7_-Sph, and ^2^H_7_-ceramide levels were measured using LC-MS/MS. Values represent the means ± SD of the lipid amounts obtained from three independent experiments (∗*p* < 0.05; ∗∗*p* < 0.01; Student's *t* test). *C*, total RNA prepared from WT and *PLPP1–3* TKO HAP1 cells was subjected to quantitative real-time RT-PCR using specific primers for ceramide synthases (*CERS1–6*) and *GAPDH*. Values represent the means ± SD and indicate the quantities relative to that of *GAPDH* from three independent reactions. Cer, ceramide; nd, not detected; PLPP, phospholipid phosphatase; S1P, sphingosine-1-phosphate; Sph, sphingosine.

Uptake of S1P containing seven deuterium (^2^H_7_) atoms was compared between WT and TKO cells. After incubating the cells with ^2^H_7_-S1P for 2 h, the medium and cells were separated by centrifugation, and lipids were extracted from each fraction. More abundant ^2^H_7_-S1P was present in both the medium and the cell fractions of TKO cells (2.7-fold in both fractions) than in WT cells (Fig. 3*A*). On the other hand, the amount of ^2^H_7_-Sph, the product of PLPPs, in the cell fraction of WT cells was about 6.8 times higher than in TKO cells. Since the ^2^H_7_-S1P and ^2^H_7_-Sph detected in the cell fraction possibly include not only those transported into cells but also those attached to the cell surface, the amounts of ^2^H_7_-S1P/^2^H_7_-Sph detected in the cell fraction might not accurately represent uptake. We considered that it would be useful to measure the amounts of the ^2^H_7_-S1P metabolite ^2^H_7_-ceramides, which are produced only within cells, as indicators of the actual amount of ^2^H_7_-S1P transported into cells. The amounts of ^2^H_7_-ceramides were reduced to 17% in TKO cells compared with WT cells. This result indicates that the uptake of ^2^H_7_-S1P indeed decreased in TKO cells.

Next, we examined the uptake of ^2^H_7_-Sph. There was no significant difference between the WT and TKO cells in the amounts of ^2^H_7_-Sph in the medium fraction or in the cell fraction (Fig. 3*B*). As in the case of ^2^H_7_-S1P, the ^2^H_7_-Sph recovered in the cell fraction could be a mixture of that attached to the cell surface and that actually imported into the cells. Therefore, we also measured the amounts of ^2^H_7_-Sph metabolites, ^2^H_7_-S1P and ^2^H_7_-ceramides, which are produced only within cells, as indicators of ^2^H_7_-Sph transported into cells. The amount of ^2^H_7_-S1P in TKO cells was similar to that in WT cells. In contrast, the amounts of ^2^H_7_-ceramides were 1.4 times higher in TKO cells than in WT cells. Since S1P is abundant in the serum in the medium used for growth, it is expected that S1P is constantly taken up into cells in a PLPP-dependent manner and used for sphingolipid synthesis under normal culture conditions. We speculate that the impaired uptake of S1P in TKO cells caused a difference in sphingolipid metabolism compared with WT cells, leading to increased ^2^H_7_-ceramide production. The lack of reduction of ^2^H_7_-ceramides in TKO cells indicates that ceramide synthase activity is normal in TKO cells. We also examined the expression levels of ceramide synthases (*CERS1–6*) using quantitative real-time RT-PCR. In both HAP1 WT and TKO cells, *CERS1*, *CERS2*, *CERS4*, and *CERS6* mRNAs were expressed, and there were almost no differences in expression levels between the cell lines (Fig. 3*C*). Therefore, the decreased ^2^H_7_-ceramide production in TKO cells in the ^2^H_7_-S1P labeling experiment was attributed to the reduction in ^2^H_7_-S1P uptake, not to decreased ceramide synthase activity. In conclusion, although most extracellular S1P is taken up into cells after dephosphorylation by PLPPs, an S1P uptake pathway independent of PLPPs is also present.

### A direct S1P transport pathway exists

As a PLPP-independent S1P uptake pathway, two possibilities were considered: import after dephosphorylation by another phosphatase or direct import. In the former case, one possibility is that S1P is endocytosed and dephosphorylated by lysosomal phosphatases. Here, we investigated the latter possibility by subjecting WT and TKO cells to [^32^P]S1P labeling and [^32^P]orthophosphoric acid (Pi) labeling experiments. [^32^P]Pi-labeled lipids must represent *de novo* phospholipid synthesis (Fig. 1). If all [^32^P]S1P is dephosphorylated and then taken up by cells, the pattern of lipids labeled by [^32^P]S1P and [^32^P]Pi should be the same. On the other hand, if [^32^P]S1P is directly imported, some of the imported [^32^P]S1P would be metabolized to [^32^P]phosphoethanolamine (and unlabeled *trans-*2-hexadecenal) by S1P lyase and then metabolized to [^32^P]PE *via* [^32^P]CDP-ethanolamine (13, 17, 18) (Fig. 1). A fraction of the generated [^32^P]PE could be metabolized to [^32^P]phosphatidylserine (PS) and further to [^32^P]phosphatidylcholine (PC) by base-exchange reactions (18, 42).

**Figure 4:**
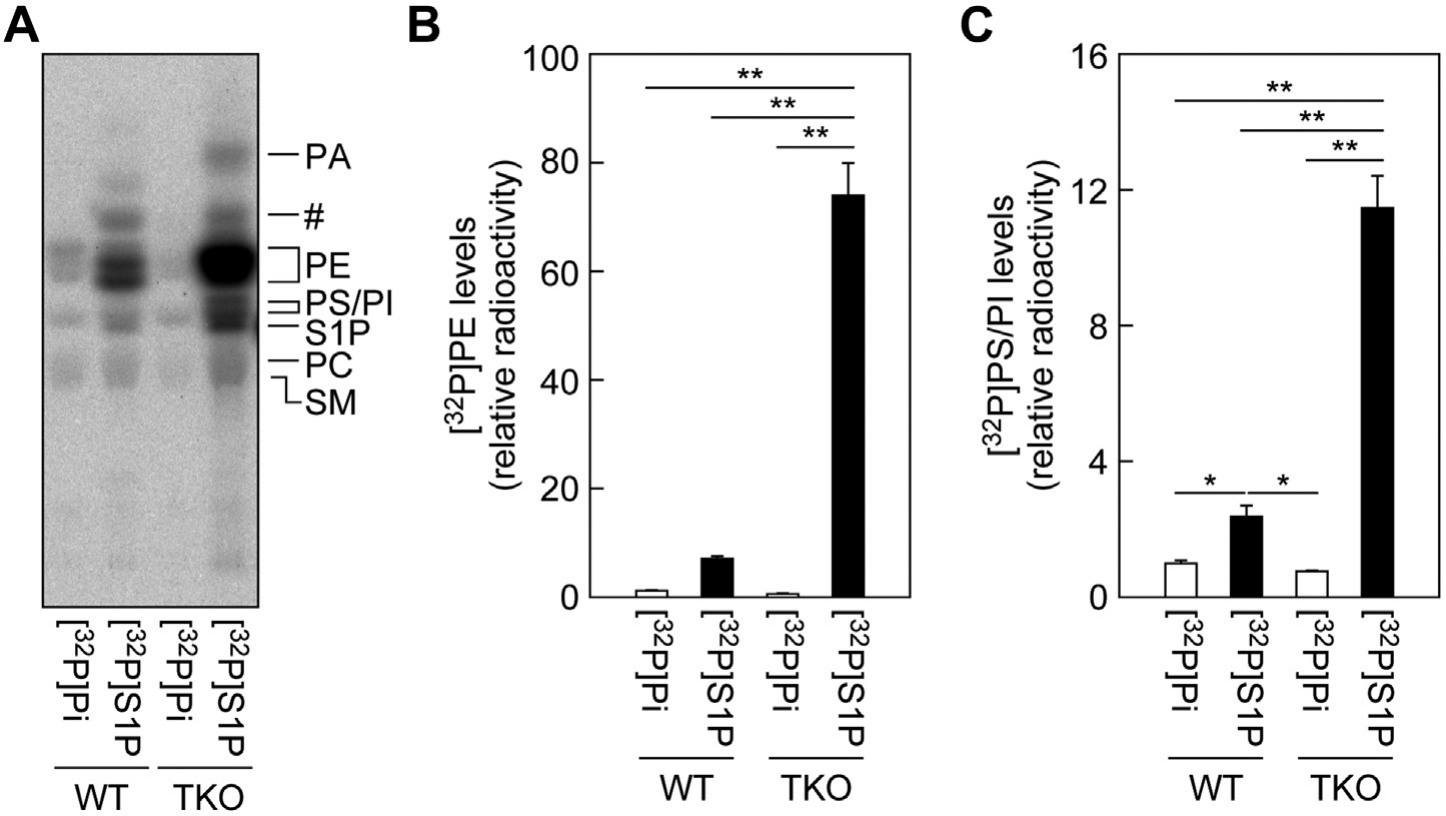
**A fraction of the extracellular S1P is imported directly into cells.***A–C*, WT and *PLPP1–3* TKO HAP1 cells were labeled with 0.2 μCi [^32^P]Pi or 0.2 μCi [^32^P]S1P for 3 h in the presence of 1% bovine serum albumin. After washing the cells, lipids were extracted, separated by TLC, and detected (*A*) and quantified (*B* and *C*) using an imaging analyzer BAS2500. Values represent the means ± SD of the radioactivity of [^32^P]PE (*B*) or [^32^P]PS/PI (*C*) relative to that in respective [^32^P]Pi-labeled WT cells, from three independent experiments (∗*p* < 0.05; ∗∗*p* < 0.01; Tukey's test). PE, phosphatidylethanolamine; Pi, orthophosphoric acid; PI, phosphatidylinositol; PS, phosphatidylserine; SM, sphingomyelin; S1P, sphingosine-1-phosphate; TKO, triple KO; #unidentified lipid.

In both WT and TKO cells, similar amounts of [^32^P]PE, [^32^P]PS, [^32^P]phosphatidylinositol (PI), [^32^P]PC, and [^32^P]sphingomyelin were labeled by [^32^P]Pi (Fig. 4*A*). PE was the lipid most intensively labeled by [^32^P]S1P in both WT and TKO cells. In WT cells, the levels of [^32^P]PE produced by [^32^P]S1P labeling tended to be higher compared with [^32^P]Pi labeling, and these levels were much higher in TKO cells (126.6-fold; [^32^P]Pi labeling *versus* [^32^P]S1P labeling) (Fig. 4*B*). In the [^32^P]S1P labeling, the amount of [^32^P]PE in TKO cells was 10.5-fold higher than in WT. [^32^P]PS/[^32^P]PI levels were also higher in [^32^P]S1P-labeled cells than in [^32^P]Pi-labeled cells (WT, 2.4-fold; and TKO, 14.7-fold), and the levels were higher in TKO cells than in WT cells by 4.8-fold (Fig. 4*C*). Note that PS and PI cannot be separated under the TLC conditions used, but most of the increase should be in [^32^P]PS, considering the metabolic pathway of PE (Fig. 1). Increases in [^32^P]PC and [^32^P]PA levels were also observed in [^32^P]S1P-labeled TKO cells compared with the corresponding WT cells (Fig. 4*A*). Most of the [^32^P]PA seemed to be produced *via* degradation of [^32^P]PE by phospholipase D. These results indicate that some S1P is directly imported into cells without dephosphorylation. Importantly, this pathway works even in WT cells, although its action became more apparent in the absence of *PLPP1–3*.

### SPNS2 and MFSD2B exhibit S1P import activity

**Figure 5:**
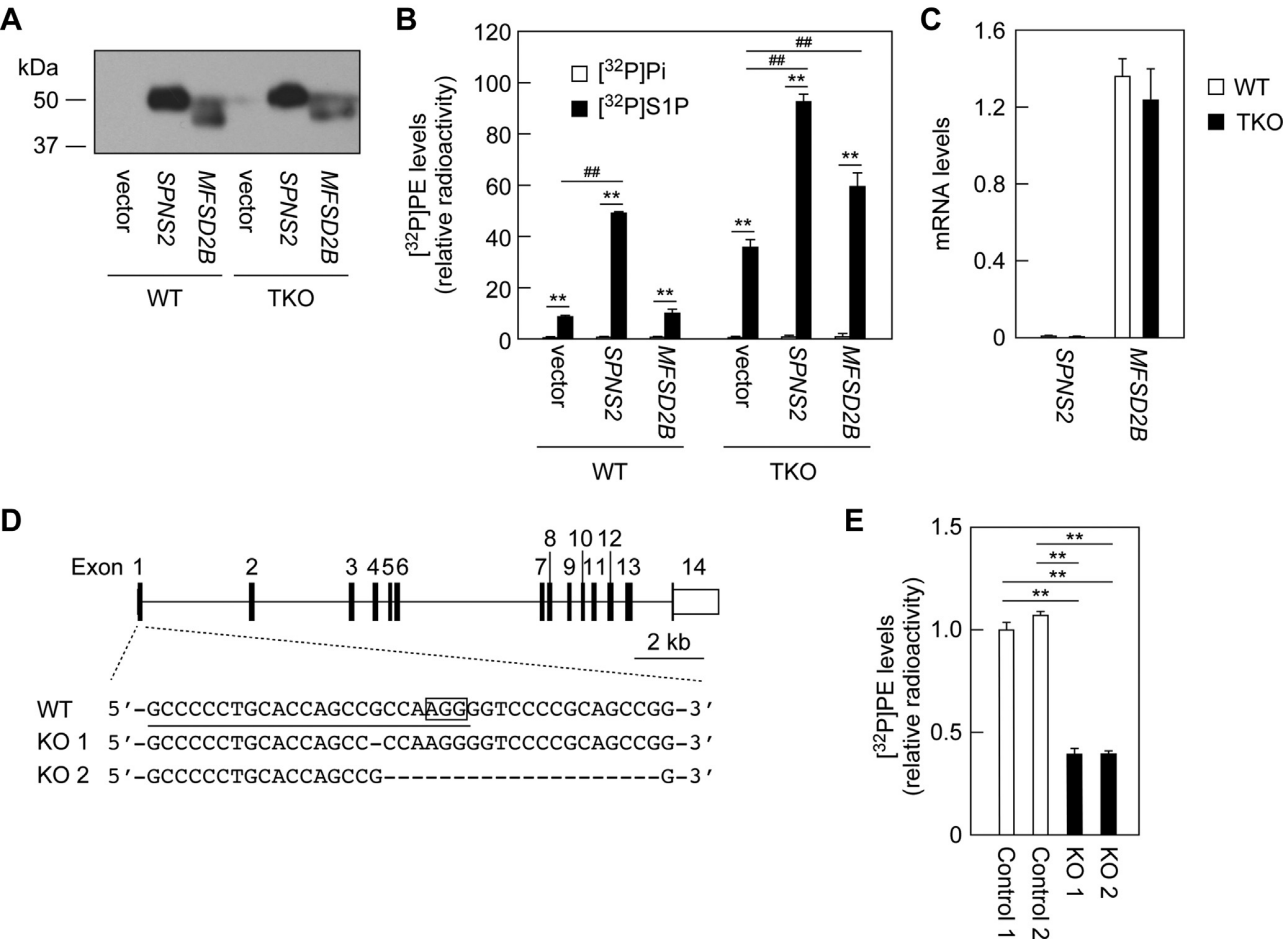
**SPNS2 and MFSD2B exhibit S1P uptake activity.***A* and *B*, WT and *PLPP1–3* TKO HAP1 cells were transfected with pCE-puro 3×FLAG-1 (vector), pHG7 (*3×FLAG-SPNS2*), or pHG8 (*3×FLAG-MFSD2B*) plasmid. Twenty-four hours after transfection, cells were treated with 2 μg/ml puromycin for 3 h to select the plasmid-introduced cells. The cells were subcultured onto new dishes and were grown for 21 h in the presence of puromycin and then for 24 h in the absence of puromycin. Cells were labeled with 0.2 μCi [^32^P]Pi or [^32^P]S1P for 3 h in the presence of 1% bovine serum albumin, and proteins and lipids were prepared from the cells. *A*, protein samples prepared from [^32^P]Pi-labeled cells were separated by SDS-PAGE and subjected to immunoblotting with anti-FLAG antibody. *B*, lipids were separated by TLC, and [^32^P]PE levels were quantified using an imaging analyzer BAS2500. Values represent the means ± SD of the radioactivity relative to that in [^32^P]Pi-labeled WT cells harboring the vector, from three independent experiments (∗∗*p* < 0.01, [^32^P]Pi-labeled *versus* [^32^P]S1P-labeled, Student's *t* test; ^##^*p* < 0.01, vector *versus SPNS2* or *MFSD2B*, Dunnett's test). *C*, total RNA prepared from WT and TKO HAP1 cells was subjected to quantitative real-time RT-PCR using specific primers for *SPNS2*, *MFSD2B*, and *GAPDH*. Values presented are means ± SD and indicate the quantities relative to that of *GAPDH* from three independent reactions. *D*, *MFSD2B* KO cells were constructed from TKO HAP1 cells using the CRISPR/Cas9 system. The genome structure of *MFSD2B* (coding sequences, *black boxes*; untranslated regions, *white boxes*), the location and types of mutations, a protospacer adjacent motif (*enclosed*), and the guide RNA sequence (*underlined*) are shown. *E*, *MFSD2B* WT (control 1 and 2) and KO cells (KO 1 and KO 2) were labeled with 0.2 μCi [^32^P]S1P for 1 h in the presence of 1% bovine serum albumin. Lipids were separated by TLC, and [^32^P]PE levels were quantified using an imaging analyzer BAS2500. Values presented are means ± SD of radioactivity levels relative to those in control clone 1, from three independent experiments (∗∗*p* < 0.01; Tukey's test). MFSD2B, major facilitator superfamily domain containing 2B; PE, phosphatidylethanolamine; Pi, orthophosphoric acid; PLPP, phospholipid phosphatase; S1P, sphingosine-1-phosphate; SPNS2, sphingolipid transporter 2; TKO, triple KO.

Although SPNS2 and MFSD2B have been identified as S1P export transporters (26, 27, 28), the transporters that import S1P are unknown. Among transporters, channel-type transporters move substances passively: the direction of transport depends on the concentration difference inside and outside the membrane. It is not known whether SPNS2 and MFSD2B are pump- or channel-type transporters. We investigated whether SPNS2 or MFSD2B could import S1P as channel-type transporters. WT and TKO cells overexpressing 3×FLAG-tagged SPNS2 or MFSD2B were labeled with [^32^P]S1P or [^32^P]Pi. The expression levels of SPNS2 were slightly higher than those of MFSD2B (Fig. 5*A*). When SPNS2 was overproduced in WT cells, S1P uptake (as determined by [^32^P]PE amount) was increased by 5.4-fold compared with the vector control (Fig. 5*B*). Overexpression of MFSD2B had no effect in WT cells. On the other hand, S1P uptake activity was increased by overexpression of either SPNS2 or MFSD2B (SPNS2, 2.6-fold; MFSD2B, 1.6-fold) in TKO cells. Thus, overexpression of SPNS2 enhanced S1P uptake more strongly than that of MFSD2B. However, taking into account the difference in their expression levels (Fig. 5*A*), it is likely that the S1P uptake activities of SPNS2 and MFSD2B are comparable. [^32^P]Pi uptake was not affected by overexpression of SPSN2 or MFSD2B in either cells. These results indicate that both SPNS2 and MFSD2B are channel-type transporters that can transport S1P inward.

In both WT and TKO HAP1 cells, the expression levels of *SPNS2* were low, whereas those of *MFSD2B* were high (Fig. 5*C*). Next, to investigate the involvement of *MFSD2B* in S1P uptake in TKO cells, we disrupted *MFSD2B* in TKO cells using the CRISPR/Cas9 system. We obtained two clones with deletions of 1 and 20 bp, respectively, in exon 1 of *MFSD2B* (Fig. 5*D*). These clones and the control clones were then subjected to a [^32^P]S1P labeling assay. The levels of [^32^P]PE produced in *MFSD2B* KO clones were approximately 40% of those of the controls (Fig. 5*E*). This result suggests that *MFSD2B* is involved in S1P uptake in HAP1 TKO cells.

### The MEDEP-E14 erythroid cells show high S1P uptake activity

**Figure 6:**
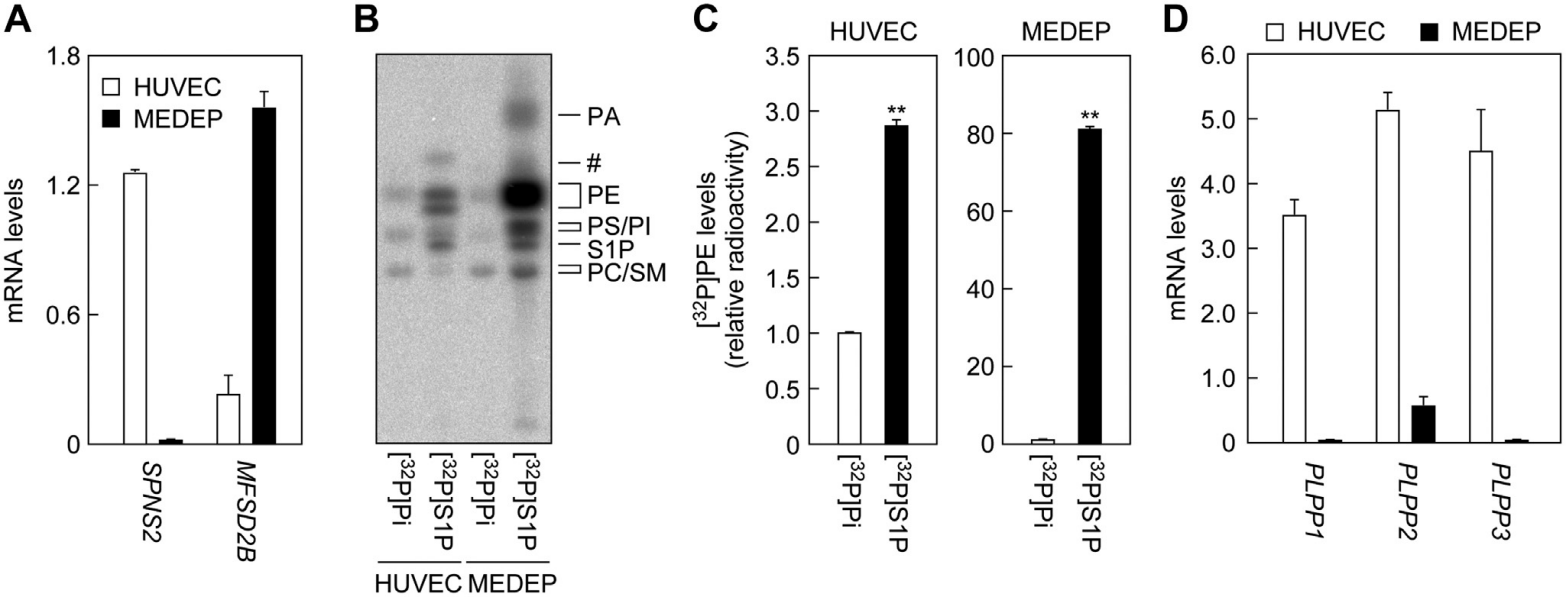
**MEDEP-E14 cells exhibit high S1P import activity.***A*, total RNA prepared from HUVECs and MEDEP-E14 cells was subjected to quantitative real-time RT-PCR using specific primers for *SPNS2*, *MFSD2B*, and *GAPDH*. Values presented are means ± SD and indicate quantities relative to that of *GAPDH* from three independent reactions. *B* and *C*, HUVECs and MEDEP-E14 cells were labeled with 0.2 μCi [^32^P]Pi or 0.2 μCi [^32^P]S1P for 3 h in the presence of 1% bovine serum albumin. Lipids were extracted and separated by TLC, followed by detection (*B*) and quantification (*C*) using an imaging analyzer BAS2500. Values represent the means ± SD of the radioactivity of [^32^P]PE relative to that in [^32^P]Pi-labeled HUVECs from three independent experiments (∗∗*p* < 0.01; Student's *t* test). *D*, total RNA prepared from HUVECs and MEDEP-E14 cells was subjected to quantitative real-time RT-PCR using specific primers for *PLPP1–3* and *GAPDH*. Values presented are means ± SD and indicate quantities relative to that of GAPDH from three independent reactions. HUVECs, human umbilical vein endothelial cells; MEDEP, MEDEP-E14; MFSD2B, major facilitator superfamily domain containing 2B; PE, phosphatidylethanolamine; Pi, orthophosphoric acid; PLPP, phospholipid phosphatase; SM, sphingomyelin; S1P, sphingosine-1-phosphate; SPNS, sphingolipid transporter; #unidentified lipid.

SPNS2 and MFSD2B are expressed in endothelial cells and erythrocytes, respectively (27, 28, 29, 30). We next conducted a [^32^P]S1P labeling assay on human umbilical vein endothelial cells (HUVECs) and erythroid cells (mouse ES cell-derived erythroid progenitor line [MEDEP]-E14). Since erythrocytes do not synthesize phospholipids because of their lack of intracellular organelles, they cannot be subjected to our assay system, in which [^32^P]S1P uptake is evaluated by the production of [^32^P]phospholipids (especially [^32^P]PE). Therefore, MEDEP-E14 cells, which are known to express MFSD2B (28), were used instead of erythrocytes. We used quantitative real-time RT-PCR to confirm that SPNS2 and MFSD2B were highly expressed in HUVECs and MEDEP-E14 cells, respectively (Fig. 6*A*). In HUVECs, the amount of [^32^P]PE generated by [^32^P]S1P labeling was 2.9-fold higher compared with [^32^P]Pi labeling (Fig. 6, *B* and *C*). In MEDEP-E14 cells, [^32^P]PE production by [^32^P]S1P labeling was much more prominent, 63.3 times that by [^32^P]Pi labeling. Substantial amounts of [^32^P]PA and [^32^P]PS/PI were also produced in [^32^P]S1P-labeled MEDEP-E14 cells. Thus, endothelial cells, and erythroid cells in particular, can import exogenous S1P directly.

Next, we examined the expression levels of *PLPP1–3* mRNAs in HUVECs and MEDEP-E14 cells using quantitative real-time RT-PCR. *PLPP1–3* were highly expressed in HUVECs, whereas their expression levels in MEDEP-E14 cells were low (Fig. 6*D*). This suggests that the lower uptake of S1P in HUVECs than in MEDEP-E14 cells may be due to their high levels of PLPP activity, which degrades S1P before it is taken up by the cells.

### Albumin inhibits S1P uptake

**Figure 7:**
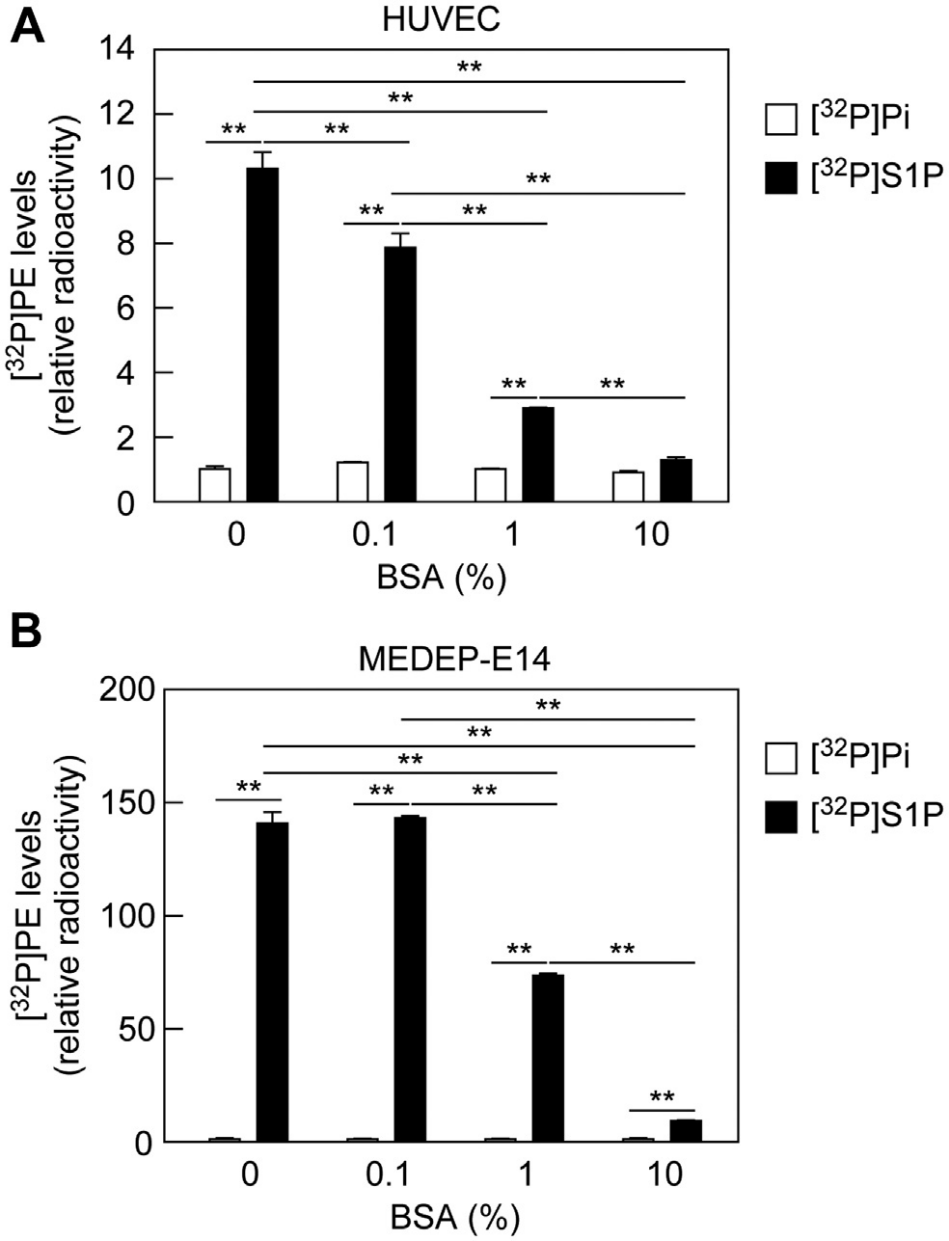
**Bovine serum albumin (BSA) inhibits S1P uptake.***A* and *B*, HUVECs (*A*) and MEDEP-E14 cells (*B*) were labeled with 0.2 μCi [^32^P]Pi or 0.2 μCi [^32^P]S1P for 3 h in the presence of 0, 0.1, 1, or 10% BSA. Lipids were extracted and separated by TLC, and [^32^P]PE levels were quantified using an imaging analyzer BAS2500. Values represent the means ± SD of the radioactivity relative to that in [^32^P]Pi-labeled cells without BSA treatment from three independent experiments (∗∗*p* < 0.01; Tukey's test). HUVEC, human umbilical vein endothelial cell; MEDEP, mouse ES cell-derived erythroid progenitor line; PE, phosphatidylethanolamine; Pi, orthophosphoric acid; S1P, sphingosine-1-phosphate.

Plasma S1P exists in a state where it is bound to albumin or HDL (31, 33). Most of the aforementioned experiments were conducted in the presence of 1% bovine serum albumin (BSA). We next examined the effect of BSA concentration on S1P uptake. [^32^P]S1P was added to the medium of HUVECs or MEDEP-E14 cells in the presence of 0% to 10% BSA. In both cells, the amounts of [^32^P]PE produced decreased in the presence of BSA in a concentration-dependent manner (Fig. 7, *A* and *B*). In contrast, BSA had little effect on [^32^P]Pi uptake. These results indicate that BSA competes with the S1P transporters for the binding of S1P. In conclusion, the uptake efficiency of S1P by the transporters differs depending on whether S1P is in a free state or is albumin bound.

## Discussion

S1P is a lipid mediator that plays a particularly important role in the vascular and immune systems (1, 2, 3). So far, regarding the clearance of S1P in plasma, it has been known that S1P is dephosphorylated by PLPPs present on the cell surface of cells in contact with plasma and is then taken up into cells (41). However, the existence of a direct S1P uptake pathway without dephosphorylation remained unclear. In the present study, we found that a direct S1P uptake pathway exists (Fig. 4) and is particularly active in erythroid cells (Fig. 6).

Here, we revealed that the erythroid MEDEP-E14 cells exhibit a high S1P uptake activity. We did not investigate the S1P uptake activity of erythrocytes, which cannot synthesize phospholipids, because of the limitation of our assay system, which uses ^32^P-labeled phospholipids as indicators of S1P uptake. However, it has been reported that erythrocytes show a high S1P uptake activity (34). In that article, erythrocytes treated with S1P were centrifuged and separated into supernatant and erythrocyte fractions, and the amount of S1P in each fraction was measured. Strictly speaking, in that experiment, S1P imported into erythrocytes was indistinguishable from S1P attached to the erythrocyte surface, and both should have been recovered in the erythrocyte fraction as a mixture. However, considered in the light of our results, it is likely that most of the S1P in the erythrocyte fraction was actually imported into erythrocytes. The previous article proposed that erythrocytes act as a reservoir that regulates plasma S1P levels (34), and our current study supports this idea.

We have shown that SPNS2 and MFSD2B, which have been considered to be S1P-exporting transporters (26, 27, 28), are also involved in inward S1P transport (Fig. 5). Our findings suggest that they are not pump-type transporters, which only transport substances in one direction using energy, but are channel-type transporters, which can transport substances in both directions, depending on the concentration gradient. However, the bidirectional transport of S1P by SPNS2 and MFSD2B needs to be proven in future experiments involving proteoliposomes.

Plasma S1P levels are high, whereas intracellular S1P levels are low because of the high activity of intracellular degradation enzymes (S1P lyase and phosphatases) (20, 21). However, most S1P in plasma is bound to albumin or HDL, and free S1P levels are low (31, 33). Therefore, the concentration of free S1P seems to be higher in S1P-producing cells than in plasma, leading to a more predominant release of S1P from the cells than uptake under normal conditions. In contrast, in a situation where a large amount of S1P is released from activated platelets into plasma because of damaged blood vessels, the locally increased S1P may be taken up and removed by erythrocytes.

The addition of BSA inhibited the uptake of S1P into cells (Fig. 7). This inhibitory effect is likely because of competition between the S1P transporters and BSA for S1P binding. We could not examine the effects of HDL on S1P uptake, since we could not prepare [^32^P]S1P-bound HDL. Considering that HDL also has the ability to bind to S1P, HDL is likely to inhibit S1P uptake in the same manner as BSA. Albumin concentration in human plasma is 4% to 5% (43). MEDEP-E14 cells exhibited S1P uptake activity even in the presence of 10% BSA (Fig. 7*B*). Therefore, erythrocytes may import S1P under physiological conditions. We speculate that the S1P released from platelets upon vascular injury is in free state and more easily taken up by nearby erythrocytes or endothelial cells before binding to albumin or HDL. When overexpressed in cells, SPNS2 showed high S1P uptake activity (Fig. 5*B*). However, the S1P uptake activity of HUVECs, in which *SPNS2* and *PLPP1–3* were highly expressed, was low (Fig. 6). Thus, S1P uptake is regulated by the expression levels of both S1P transporters and PLPPs.

Plasma S1P concentration has previously been considered to be maintained by the balance between release from S1P-producing cells (mainly erythrocytes but also endothelial cells) and degradation by PLPPs on the surface of cells in contact with plasma. In addition to these, we here found a new regulatory mechanism for plasma S1P concentration: an S1P uptake pathway that occurs without S1P dephosphorylation. Since the S1P transporters SPNS2 and MFSD2B are expressed almost exclusively in endothelial cells, erythrocytes, and platelets (27, 28, 29, 30), this pathway may have little effect on S1P signaling *via* S1PRs in most cells. However, further studies are needed to elucidate the pathophysiological role of S1P uptake by S1P transporters.

## Experimental procedures

### Cells, growth conditions, and transfection

Myelogenous leukemia-derived, near-haploid human HAP1 cells (44) were purchased from the American Type Culture Collection and cultured in Iscove's modified Dulbecco's medium (Thermo Fisher Scientific) containing 10% fetal bovine serum (FBS), 100 units/ml penicillin, and 100 μg/ml streptomycin (Merck). HUVECs were purchased from Cell Applications and grown in endothelial cell basal medium 2 supplemented with SupplementPack Endothelial Cell GM2 (PromoCell) on collagen-coated dishes. MEDEP-E14 cells (45) were obtained from RIKEN BioResource Research Center and grown in Iscove's modified Dulbecco's medium containing 15% FBS, 100 units/ml penicillin, 100 μg/ml streptomycin, 1× ITS Liquid Media Supplement (Merck), 0.45 mM α-monothioglycerol (Merck), 50 mg/ml ascorbic acid (Merck), and 3 units/ml human erythropoietin (Kyowa Kirin). Cells were cultured at 37 °C under 5% CO_2_. Transfections were performed using the reagents Lipofectamine and PLUS (Thermo Fisher Scientific), according to the manufacturer's instructions.

### Plasmids

The human *SPNS2* and *MFSD2B* genes were amplified from human kidney and placenta complementary DNA (Takara Bio), respectively, by PCR using the following primers: *SPNS2*, 5'-AGATCTATGATGTGCCTGGAATGCGCCTCGG-3' and 5'-TCAGACTTTCACAGATGCGGGCGGC-3'; and *MFSD2B*, 5'-GGATCCATGGCGGCGCCCCCTGCAC-3' and 5'-TTAGGCCAGGCTGTAGCTGGTCCTC-3'. The amplified genes were first cloned into the TA cloning vector pGEM-T Easy (Promega) and then transferred to pCE-puro-3×FLAG-1, which is a vector for the expression of a protein with a 3×FLAG tag at the N terminus (46) and has a puromycin-resistant gene, producing pHG7 (*3×FLAG-SPNS2*) and pHG8 (*3×FLAG-MFSD2B*) plasmids. The pCE-puro LPP1a-hemagglutinin (HA), pCE-puro LPP2-HA, and pCE-puro LPP3-HA plasmids, encoding HA-tagged *PLPP1*, *PLPP2*, and *PLPP3*, respectively, have been described previously (47).

### Construction of KO cells

To construct *PLPP1–3* TKO cells, the CRISPR/Cas9 targeting plasmid was constructed employing the Golden Gate assembly method using the pX330A/S vectors and paired oligonucleotides for each guide RNA (*PLPP1*, 5'-CAAGACGCGGCTGCCGTACGGTTTT-3' and 5'-CGTACGGCAGCCGCGTCTTGCGGTG-3'; *PLPP2*, 5'-GTGAACGCCCCGTACAAGCGGTTTT-3' and 5'-CGCTTGTACGGGGCGTTCACCGGTG-3'; *PLPP3*, 5'-GTACGACAAAGCGATCGTCCGTTTT-3' and 5'-GGACGATCGCTTTGTCGTACCGGTG-3') as described previously (48). HAP1 cells were transfected with the resulting CRISPR/Cas9 targeting plasmid together with the puromycin resistant gene-containing vector pCE-puro 3×FLAG-1, and, 24 h after transfection, they were subjected to puromycin (2 μg/ml) treatment for 3 days. Cells were then subcultured on new dishes at low cell density to form colonies. Genomic DNA was prepared from the obtained clones. Each of the *PLPP1–3* genes was amplified by PCR, and the mutations were examined by DNA sequencing. Among the obtained clones, those with mutations in all *PLPP1–3* genes were used for further study. The mutations in the selected TKO cells were as follows: 1-bp insertion in *PLPP1*, 11-bp deletion in *PLPP2*, and 5-bp deletion in *PLPP3*.

*MFSD2B*-deficient TKO cells were generated using the pX330S vector, oligonucleotide pairs (5'-GCCCCCTGCACCAGCCGCCAGTTTT-3' and 5'-TGGCGGCTGGTGCAGGGGGCCGGTG-3'), and HAP1 TKO cells, as described above. We obtained two clones that contain deletions of 1 and 20 bp, respectively, in exon 1 of *MFSD2B*. Two control clones were also obtained by transfection of TKO cells with the empty pX330S vector.

### Quantitative real-time RT-PCR

Total RNA was prepared from HAP1 cells using the NucleoSpin RNA II Kit (Machery-Nagel), according to the manufacturer's instructions. Quantitative real-time RT-PCR was performed using the One Step SYBR PrimeScript RT-PCR Kit II (Takara Bio), according to the manufacturer's instructions. The primer pair for *PLPP1* was 5'-CAGGCCACTCTTCGTTTTCCATGTA-3' and 5'-GCAGAGTTGTATGAGAGTCCTCCTC-3'; for *PLPP2*, 5'-TGTCTTTCTACTCGGGACACTCTTC-3' and 5'-CCAGCTCCTCCTCCTTCAGACAGTG-3'; for *PLPP3*, 5'-CTATACCTGCAGGCCCGCTTCACTT-3' and 5'-CATCATGTTGTGGTGATTGTTCCTG-3'; for *CERS1*, 5'-CTGGCGCAAGGACTCGGTGG-3' and 5'-ATTGTGGTACCGGAAGGCG-3'; for *CERS2*, 5'-GCTGGAGTCAGCCAAGATGT-3' and 5'-AGGATCCAGAAGGGCAGGAT-3'; for *CERS3*, 5'-GTTTAGGAGTCGGCGGAATCAAG-3' and 5'-AAACGCAATTCCAGCAACAGT -3'; for *CERS4*, 5'-GCAGTATCAGCAAGTGTGCG-3' and 5'-GTGGGAAAGAGGACCAGTCG-3'; for *CERS5*, 5'-ATCTTCTTCGTGAGGCTG-3' and 5'-ATGTCCCAGAACCAAGGT-3'; for *CERS6*, 5'-ATCAGGAGAAGCCAAGCACG-3' and 5'-AGTAGTGAAGGTCAGTTGTG-3'; for *SPNS2*, 5'-ATGGCTCCGAGATATGAAGGC-3' and 5'-CCAGAAATCCCGTAAAGCAGG-3'; for *MFSD2B*, 5'-GGCCAGGCCTGGAGACCATCTTCTA-3' and 5'-GAGGCGTCCCGACTGGGTGTCTTTG-3'; and for *GAPDH*, 5'-GAACGGGAAGCTCACTGGCATGGCC-3' and 5'-TGTCATACCAGGAAATGAGCTTGAC-3'.

### ^2^H_7_-Sph and ^2^H_7_-S1P labeling assays

^2^H_7_-S1P (Avanti Polar Lipids) and ^2^H_7_-Sph (Avanti Polar Lipids) were dissolved in ethanol to a concentration of 1 mM. Cells in 6-well dishes were incubated with 1 ml of medium without FBS or antibiotics for 1 h, and then the medium was replaced with one without FBS or antibiotics but containing 3.5% BSA (fatty acid free; catalog no. 6003; Merck) and 5 μM ^2^H_7_-S1P or ^2^H_7_-Sph. After incubation for 2 h, the medium was collected. The cells were washed twice with 1 ml of PBS, suspended in 300 μl of PBS, detached from the dishes using a scraper, centrifuged, and suspended in 100 μl of PBS. Lipids were extracted from the medium and cell fractions (both 100 μl) using the Bligh–Dyer method (49) with slight modification. Samples were successively mixed with 375 μl of chloroform/methanol/12 M formic acid (100:200:1, v/v), 125 μl of CHCl_3_, and 125 μl of 1% KCl, and phases were separated by centrifugation (2600*g*, room temperature, 3 min) into organic and aqueous phases. The aqueous phase was then subjected to a second phase separation by mixing it with 450 μl of chloroform and following with centrifugation. The resulting organic phase was pooled with the previous one and dried. Lipids were suspended in 100 μl of chloroform/methanol (1:2, v/v) and subjected to LC-MS/MS analyses.

LC-MS/MS analyses of ^2^H_7_-S1P, ^2^H_7_-Sph, and ^2^H_7_-ceramides were performed using ultra–high performance liquid chromatography (UPLC) coupled with electrospray ionization tandem triple quadrupole mass spectrometer (Xevo TQ-S; Waters) in multiple reaction monitoring mode. ^2^H_7_-S1P and ^2^H_7_-Sph were separated by UPLC using a YMC-Triart C18 metal-free column (length, 50 mm; particle size, 1.9 μm; inner diameter, 2.1 mm; YMC) and a binary gradient system with a mobile phase A (methanol/acetonitrile/water [1:1:3, v/v] containing 5 mM ammonium sulfate, 500 mM EDTA, and 0.025% aqueous ammonia) and a mobile phase B (2-propanol containing 5 mM ammonium sulfate, 500 mM EDTA, and 0.025% aqueous ammonia) at a flow rate of 0.25 ml/min at 40 °C. Gradient conditions were as follows: 0 min, 0% B; 0 to 1 min, gradient to 50% B; 1 to 5 min, 64% B; 5 to 11 min, gradient to 95% B; 11 to 13 min, 95% B; 13 to 15 min, gradient to 0% B; 15 to 20 min, 0% B. The sample volumes injected onto the UPLC column were 5 μl. The measurements of ^2^H_7_-S1P and ^2^H_7_-Sph were performed in positive ion mode, and the *m/z* values of the respective precursor ions ([M + H]^+^) and product ions were set to mass filters Q1 (^2^H_7_-S1P, 387.2; ^2^H_7_-Sph, 307.4) and Q3 (^2^H_7_-S1P, 271.0; ^2^H_7_-Sph, 271.3), respectively. The collision energy and the sampling cone were set at 15 eV and 15 V, respectively. Data analyses were performed using MassLynx software (Waters).

LC-MS/MS analysis of ^2^H_7_-ceramides was performed using an ACQUITY UPLC CSH C18 column (length, 100 mm; particle size, 1.7 μm; inner diameter, 2.1 mm; Waters) and a binary gradient system with a mobile phase C (acetonitrile/water [3:2, v/v] containing 5 mM ammonium formate) and a mobile phase D (2-propanol/acetonitrile [9:1, v/v] containing 5 mM ammonium formate) at a flow rate of 0.3 ml/min at 55 °C. Gradient steps were as follows: 0 min, 40% D; 0 to 18 min, gradient to 100% D; 18 to 23 min, 100% D; 23 to 23.1 min, gradient to 40% D; 23.1 to 25 min, 40% D. The sample volumes injected onto the UPLC column were 5 μl of 10-fold diluted samples. Q1, Q3, and collision energies were set as shown in Table 1, and the amounts of ^2^H_7_-ceramides containing C16:0, C18:0, C20:0, C22:0, C24:0, or C26:0 fatty acid were measured in positive ion mode. The sampling cone was set to 30 V. Data analyses were performed using MassLynx software (Waters). ^2^H_7_-Ceramide amount was defined as the sum of C16:0–C26:0 ^2^H_7_-ceramide amounts.

**Table 1:** Selected *m/z* values and collision energies for ^2^H_7_-ceramide measurements in LC-MS/MS analyses

Fatty acid moiety of ^2^H_7_-ceramide species	Precursor ions (Q1)		Product ion (Q3)	Collision energy (eV)
	[M – H_2_O + H]^+^	[M + H]^+^		
C16:0	527.5	545.5	271.3	20
C18:0	555.6	573.6	271.3	20
C20:0	583.6	601.6	271.3	20
C22:0	611.6	629.6	271.3	25
C24:0	639.6	657.6	271.3	30
C26:0	667.7	685.7	271.3	30

### [^32^P]Pi and [^32^P]S1P labeling assays

[^32^P]S1P was prepared using [γ-^32^P]ATP (6000 Ci/mmol; PerkinElmer Life Sciences), Sph (Avanti Polar Lipids), and total cell lysates prepared from human embryonic kidney 293T cells overexpressing the Sph kinase SPHK2, as described previously (50), and dissolved in ethanol. [^32^P]Pi and [^32^P]S1P labeling assays were performed as follows. One hour before labeling, the medium containing FBS and antibiotics was replaced with medium devoid of them. Cells were labeled with 0.2 μCi [^32^P]Pi (phosphorus-32; 8500–9120 Ci/mmol; PerkinElmer Life Sciences) or 0.2 μCi [^32^P]S1P in the presence or the absence of fatty acid-free BSA for 1 or 3 h. After removing the medium, cells were washed twice with PBS containing 1% BSA. PBS was added to cells, and cells were detached from the dishes using a scraper, centrifuged, and suspended in PBS. Lipids were extracted as described previously, dried, suspended in chloroform/methanol (1:2, v/v), and separated by normal-phase TLC using high-performance TLC silica gel 60 plates (Merck) and 1-butanol/acetic acid/water (3:1:1, v/v) as a solvent system. ^32^P-labeled lipids were detected and quantified by an imaging analyzer BAS2500 (GE Healthcare Life Sciences).

### Immunoblotting

Proteins were recovered from the intermediate layer between the organic and aqueous layers of the phase separation in the lipid extraction described previously. Proteins were separated by SDS-PAGE and subjected to immunoblotting. Immunoblotting was performed as described previously (51), with anti-FLAG antibody M2 (1 μg/ml; Merck) as the primary antibody and the horseradish peroxidase–linked F(ab′)_2_ fragment of anti-mouse IgG (1/7500 dilution; GE Healthcare Life Sciences) as the secondary antibody. The chemiluminescent reagent, an equivalent mixture of solution A (100 mM Tris–HCl [pH 8.5], 0.4 mM *p*-coumaric acid [Merck], and 5 mM luminol [FUJIFILM Wako Pure Chemical]) and solution B (100 mM Tris–HCl [pH 8.5] and 0.04% hydrogen peroxide diluted in water), was used for detection.

## Data availability

All data generated or analyzed during this study are contained within the article.

## Conflict of interest

The authors declare that they have no conflicts of interest with the contents of this article.
